# Evaluation of the Dose Distribution Robustness for Lung Tumor Tracking in Robotic Radiotherapy Using Four-Dimensional Computed Tomography

**DOI:** 10.7759/cureus.102341

**Published:** 2026-01-26

**Authors:** Yasuhide Miyabe, Hiromu Yamanaka, Hiroto Seki, Hiromitsu Iwata, Jyunetsu Mizoe

**Affiliations:** 1 Sapporo High Functioning Radiotherapy Center, Sapporo Kojinkai Memorial Hospital, Sapporo, JPN; 2 Radiation Oncology, Nagoya City West Medical Center Hospital, Nagoya, JPN; 3 Radiation Oncology, Sapporo Kojinkai Memorial Hospital, Sapporo, JPN

**Keywords:** 4dct, cyberknife, dose distribution evaluation, dose robustness, respiratory motion, respiratory tracking

## Abstract

Respiratory motion of tumors and surrounding organs at risk (OARs) is a critical factor affecting dose distribution and treatment accuracy in radiotherapy. The CyberKnife Synchrony respiratory tracking system (Accuray Inc., Sunnyvale, CA, USA) improves targeting accuracy by estimating tumor position based on the correlation between implanted fiducial markers and external respiratory motion. However, in clinical practice, dose evaluation is commonly performed using computed tomography (CT) images acquired at a single respiratory phase, and the impact of respiratory phase variations on dose distribution has not been fully investigated.

This study is a feasibility analysis aimed at demonstrating the potential utility of four-dimensional CT (4DCT)-based simulations for evaluating dose variations associated with respiratory phase differences during Synchrony-based treatments. Two patients with lung tumors treated using CyberKnife stereotactic radiotherapy (SRT) were included in the study. Treatment plans (reference plans) were created using breath-hold CT images acquired at the end-expiratory phase. Using the same beam parameters as the reference plans, dose calculations were performed on multiple respiratory-phase images derived from free-breathing 4DCT acquired during treatment planning.

Dose-volume histogram (DVH) metrics, including clinical target volume (CTV) D99 and planning target volume (PTV) D95, as well as dose parameters for OARs, were compared with the clinical goals defined by the radiation oncologist. In Patient 1, dose reductions were observed in both the PTV and CTV at a specific inspiratory phase, resulting in the CTV dose falling below the prescribed level. In contrast, Patient 2 showed only minor dose reductions in the PTV and CTV, while prescribed dose coverage was maintained. No clinically relevant dose deterioration was observed for the OARs in either patient.

The proposed evaluation method using 4DCT demonstrated the potential to identify the influence of respiratory phase variations on dose distribution in Synchrony-based respiratory tracking radiotherapy at the treatment planning stage. This approach may serve as a supplementary tool to conventional single-phase CT-based dose evaluation for assessing plan robustness against respiratory motion.

## Introduction

The respiratory motion of tumors and organs is a key consideration in radiation therapy. To obtain computed tomography (CT) data incorporating these movements, slow-scan techniques or four-dimensional CT (4DCT) are widely used [1-3]. These methods enable target setting during treatment planning while accounting for respiratory motion. However, unwanted radiation may reach areas where the target overlaps with nearby healthy organs. To address this issue, methods such as abdominal compression to reduce respiratory motion, breath holding during deep inspiration, and beam gating at specific respiratory phases are utilized [4-7].

CyberKnife (Accuray Inc., Sunnyvale, CA, USA) is an image-guided stereotactic radiosurgery and stereotactic radiotherapy (SRT) system that enables submillimeter precision. The linear accelerator is mounted on a robotic arm that can rotate and translate, allowing a high degree of freedom in beam angles and directions. CyberKnife delivers both isocentric and nonisocentric photon beams to a target from multiple angles [8,9].

This system includes an integrated subsystem called the Synchrony respiratory tracking system (hereafter referred to as Synchrony). Synchrony estimates the tumor position by combining the motion of infrared markers placed on the thoracoabdominal surface with that of a gold fiducial marker (hereafter referred to as the marker) implanted near the tumor for tumor localization during treatment. For six-degree-of-freedom motion correction - including left-right (LR), anterior-posterior (AP), superior-inferior (SI), pitch, yaw, and roll - the use of three implanted fiducial markers is generally recommended.

The patient wears a fitted vest, and the motion of the infrared markers is continuously tracked using a stereo camera system. At the start of treatment, orthogonal X-ray images are continuously acquired to measure the internal marker position at 15 discrete time points. Based on these data, a respiratory waveform model is constructed, and the treatment beam is dynamically controlled in real time. The respiratory waveform model is periodically checked and updated during treatment by acquiring additional X-ray images at regular intervals. A detailed overview of the Synchrony system has been described in previous studies [10,11]. In addition, previous studies on treatments using the Synchrony system have primarily focused on tracking accuracy and the evaluation of geometric targeting errors [12,13].

In radiotherapy using this system, it is assumed that the motion of the implanted marker correlates with tumor motion. Therefore, dose calculation is typically performed on CT images acquired under breath-hold conditions, without explicitly considering variations in respiratory phase. However, with such treatment-planning approaches, it is not possible to fully confirm whether sufficient target coverage is maintained at each respiratory phase, which is a prerequisite for achieving a clinically acceptable cumulative dose delivery.

Therefore, this study focused on this issue and aimed to investigate the feasibility of a 4DCT-based simulation method for evaluating respiratory phase-dependent dose variations at the treatment-planning stage in Synchrony-based radiotherapy.

## Technical report

Methods

Radiotherapy was delivered using a CyberKnife M6 system. Treatment planning was performed using RayStation version 10A (RaySearch Laboratories, Stockholm, Sweden) and MultiPlan 5.3 (Accuray Inc.). Planning CT images were acquired using a SOMATOM Perspective scanner (Siemens Healthineers, Forchheim, Germany), and respiratory gating was performed with the Anzai Respiratory Gating System (AZ-733V, Anzai Medical Co. Ltd., Tokyo, Japan). At our institution, the Gold Anchor (Naslund Medical AB, Huddinge, Sweden; size: φ0.28 × 20 mm) is used as the fiducial marker. In the Synchrony system, three fiducial markers are generally required to achieve full six‑degree‑of‑freedom motion correction. However, Wu et al. proposed a treatment strategy using a single fiducial marker, and other institutions have also reported treatments performed under similar clinical conditions [14,15]. Based on these prior reports, treatments in this study were performed using a single fiducial marker.

For the treatment of lung tumors at our institution, both breath-hold CT imaging during quiet expiration, which is used as the primary CT dataset for treatment planning, and 4DCT imaging are performed. The purpose of acquiring breath-hold CT images is not limited to treatment planning; these images are also essential for generating digitally reconstructed radiographs, in which the implanted gold fiducial marker can be clearly visualized without respiratory motion artifacts. This enables reliable marker-based tracking by the Synchrony system. Four-dimensional CT images were used to evaluate the correlation between the marker and the tumor. Using the radiation treatment planning system, the center coordinates of the tumor and the marker were obtained, and the three-dimensional vector displacement of the tumor relative to the marker was calculated. At our institution, a deviation of ≤2.0 mm in the LR, AP, and SI directions is considered acceptable. The stability of the tumor marker positions was reported by Persson et al. [16] and Kupelian et al. [17]. Additionally, Seppenwoolde et al. reported that respiratory motion trajectories can exhibit loop-like paths or hysteresis curves [18]. Therefore, at our institution, one respiratory cycle - defined as the period from end-expiration (0%) to maximal inspiration (100%) and back to the next end-expiration - is analyzed by dividing it into 20 phases.

With the approval of our institutional review board (IRB No. 18000006) on October 21, 2024 (Ref No. 2024-15), this study was conducted after obtaining informed consent from the participants.

This study presents a simulation-based evaluation using two representative cases of lung metastases from adenoid cystic carcinoma originating in the right lung. The gross tumor volume (GTV), clinical target volume (CTV), and planning target volume (PTV) were defined by the oncologist based on pathology and imaging findings, with expansions of 2 mm from the GTV to the CTV, and 3 mm from the CTV to the PTV. The treatment plan was conducted using SRT, and the prescription dose was defined as the dose covering 95% of the PTV (D95), while allowing higher doses up to approximately 130% at the target center, with a total dose of 40 Gy in four fractions. Collimators with dimensions of 35 and 20 mm for Patient 1, and 25 and 15 mm for Patient 2, were selected. In both patients, the larger collimator was used in an isocentric setup to cover the PTV, and the smaller one in a non-isocentric setup to cover the marginal regions. Dose calculation was performed using the Monte Carlo algorithm.

After the oncologist’s approval, a simulation of the actual treatment was performed using the RTPS. The 4DCT images and contours at the 50% inhalation phase, maximum inhalation phase, and 50% exhalation phase were imported into the RTPS as a virtual phantom. Then, the quality assurance (QA) plan creation function in the RTPS was used to align the primary CT images with the 4DCT images, with the spine serving as the reference for six-axis corrections, including translational and rotational adjustments (pitch, roll, and yaw). The translational alignment was refined using a marker, and this alignment was visually verified by all authors. Dose calculations for the treatment beams were performed for each phase of the 4DCT images. The dose comparison was conducted using the prescribed doses and dose constraints for the PTV, CTV, and organs at risk (OARs), as defined by the radiation oncologist. As dose-volume histogram (DVH) evaluation metrics, D99 was used for the CTV, and D95 for the PTV. For the OARs, Dmax (maximum dose), the volume-based metric Vxx (volume receiving xx Gy), and the mean dose were evaluated.

Results

Table 1 and Table 2 present the dose calculation results for the reference plan and the 4DCT-based plans, relative to the clinical goals for the CTV, PTV, and OARs, established by the radiation oncologist.

**Table 1:** Dosimetric outcomes for the PTV and OARs in the reference plan and 4DCT-based plans for Patient 1 PTV, planning target volume; OARs, organs at risk; CTV, clinical target volume; 4DCT, four-dimensional computed tomography; GTV, gross tumor volume

Structure	Dose metric and clinical goal	Primary CT images	Inspiration 50%	Maximum inhalation phase	Exhalation 50%
Spinal cord	Dmax ≤ 2500 (cGy)	940.7	716	697.2	733.2
Heart	V3000 cGy ≤ 15 (cm³)	0.0	0.0	0.0	0.0
Lungs - GTV	V2000 cGy ≤ 20 (%)	2.61	2.23	2.07	2.41
	V1500 cGy ≤ 25 (%)	3.90	3.46	3.36	3.74
	Dmean ≤ 1800 (cGy)	300.0	276.0	271.0	297.0
PTV	D95 ≥ 4000 (cGy)	4046.7	3458.0	3747.2	3955.6
CTV	D99 ≥ 4000 (cGy)	4355.8	3903.6	4144.8	4285.6

**Table 2:** Dosimetric outcomes for the PTV and OARs in the reference plan and 4DCT-based plans for Patient 2 PTV, planning target volume; OARs, organs at risk; CTV, clinical target volume; 4DCT, four-dimensional computed tomography; GTV, gross tumor volume

Structure	Dose metric and clinical goal	Primary CT images	Inspiration 50%	Maximum inhalation phase	Exhalation 50%
Spinal cord	Dmax ≤ 2500 (cGy)	1281.2	1239.2	1227.2	1192.8
Heart	V3000 cGy ≤ 15 (cm³)	0.0	0.0	0.0	0.0
Lungs - GTV	V2000 cGy ≤ 20 (%)	2.39	2.70	2.43	2.67
	V1500 cGy ≤ 25 (%)	3.51	4.03	3.59	3.93
	Dmean ≤ 1800 (cGy)	299.0	314.0	300.0	306.0
PTV	D95 ≥ 4000 (cGy)	4044.7	3960.0	3959.9	4004.8
CTV	D99 ≥ 4000 (cGy)	4226.7	4121.2	4071.6	4130.8

**Figure 1:**
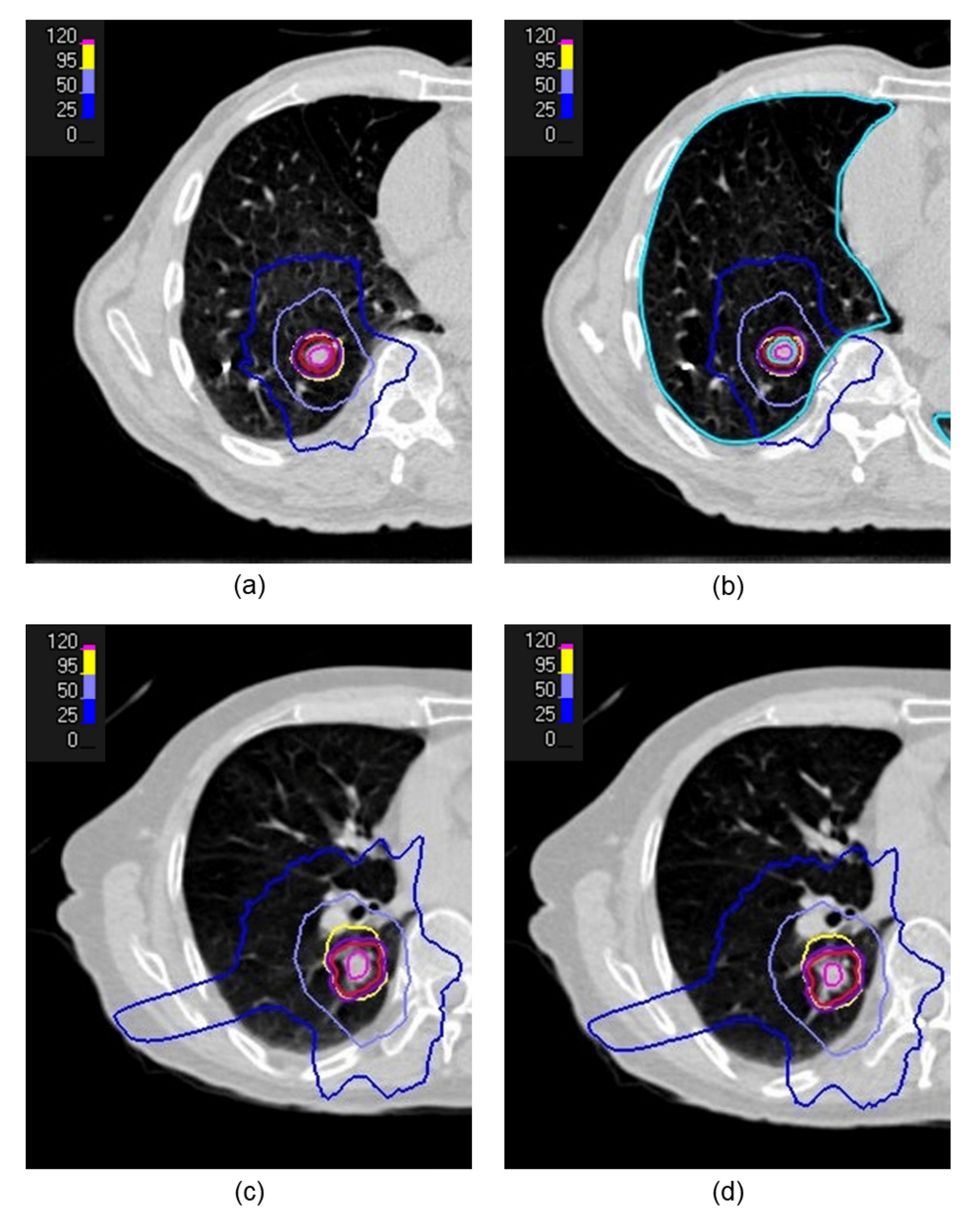
Dose distributions for Patient 1 and Patient 2 Dose distributions for Patient 1: (a) reference plan and (b) 4DCT-based plan at the 50% inspiratory respiratory phase. Dose distributions for Patient 2: (c) reference plan and (d) 4DCT-based plan at the maximum inhalation phase. The CTV is shown in red, and the PTV in dark purple. The 25%, 50%, 95%, and 120% isodose levels, relative to the prescribed dose, are shown in blue, light blue, yellow, and purple, respectively. CTV, clinical target volume; PTV, planning target volume; 4DCT, four-dimensional computed tomography

**Figure 2:**
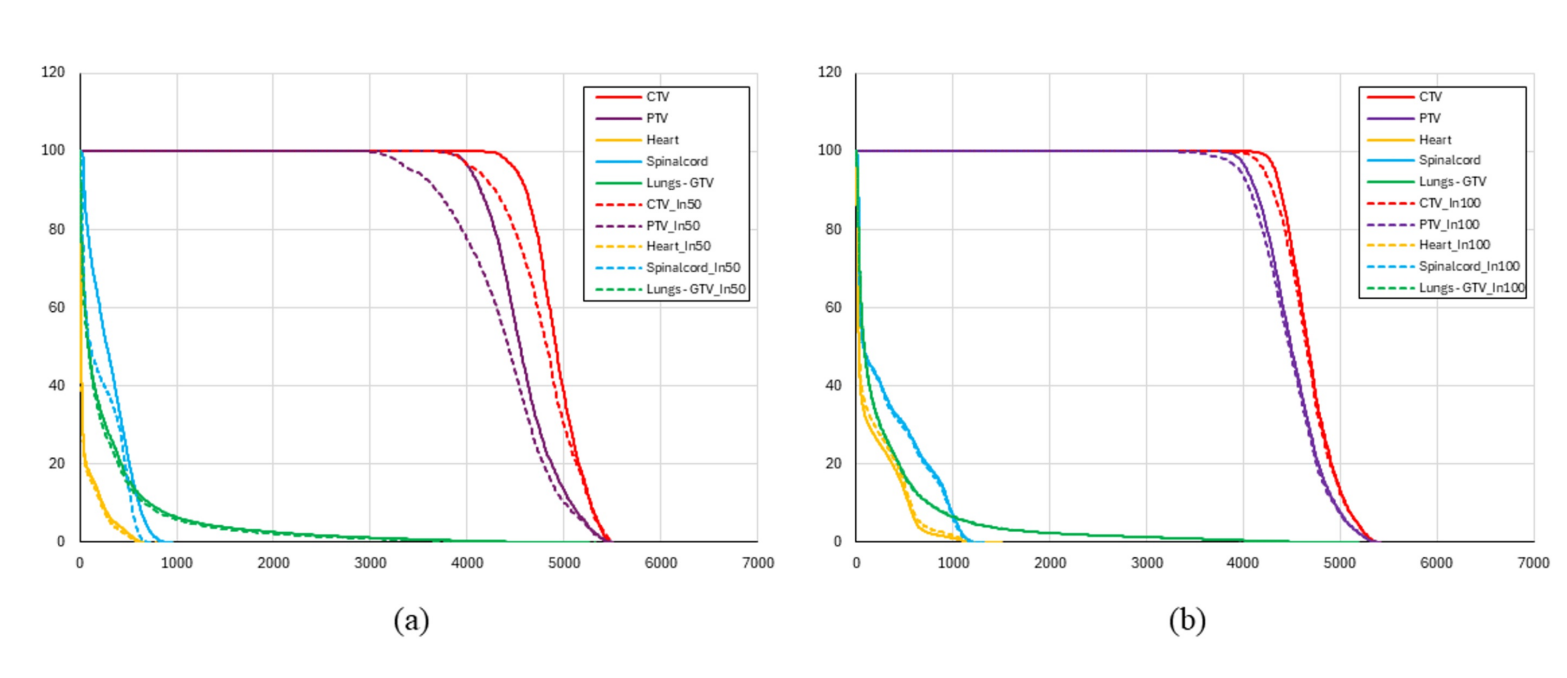
Dose-volume histograms (a) A comparison between the reference plan (solid lines) and the 4DCT-based plan (dashed lines) at the 50% inspiratory phase for Patient 1, and (b) a comparison between the reference plan (solid lines) and the 4DCT-based plan (dashed lines) at the maximum inhalation phase for Patient 2. “In50%” and “In100%” denote the 50% inspiratory phase and the maximum inhalation phase, respectively. CTV, clinical target volume; PTV, planning target volume; 4DCT, four-dimensional computed tomography.

The greatest dose difference at the CTV D99% was observed in the 50% inhalation phase for Patient 1, and at the maximum inhalation phase for Patient 2. The dose distribution maps and DVHs for the reference plan and the respiratory phase showing the largest dose differences are presented in Figure 1 and Figure 2.

In Patient 1, compared with the reference plan, a maximum dose reduction of 14.55% was observed at the PTV D95%. In addition, a 10.38% reduction at the CTV D99% was observed, resulting in the CTV dose falling below the prescription level, whereas no significant deterioration was observed in the OARs. As shown in Figures 1a-1b, the dose distribution at the 50% inspiratory phase exhibited a steeper dose gradient at the periphery of the PTV, compared with the reference plan. This spatial change was also evident in the DVH (Figure 2a), where a leftward shift of the PTV curve was observed.

In Patient 2, a 2.10% dose reduction at the PTV D95% was observed. Although a 3.67% reduction at the CTV D99% was noted, the prescription dose was still maintained, and no substantial differences were observed in the OARs. Consistent with these findings, no marked differences were observed in the dose distribution at the maximum inhalation phase, compared with the reference plan (Figures 1c-1d), and only small differences were observed in the DVH (Figure 2b).

## Discussion

In this study, we investigated a method for pre-treatment evaluation of the potential impact of respiratory phase differences on dose distribution in respiratory tracking radiotherapy, by performing simulations using 4DCT images acquired during treatment planning.

The dose variations observed in the target volumes in this study are mainly attributable to two factors. First, respiratory-phase-dependent anatomical changes, such as lung expansion and rib motion, can alter beam paths. These changes may modify tissue density and water-equivalent path length along the beam trajectories, potentially resulting in variations in dose delivery. Second, limitations of marker-based tracking should be considered. While marker tracking with the Synchrony system is effective for compensating tumor motion, it cannot fully account for the deformation of surrounding tissues caused by respiration, nor for the associated changes in beam attenuation and scatter.

In Case 1, dose reductions were observed in both the PTV and CTV. This finding is likely attributable to the steep dose gradient near the ventral margin of the PTV, such that even small differences in respiratory phase may have a substantial impact on the dose distribution. In contrast, in Case 2, the doses to both the PTV and CTV remained relatively stable. This stability may be explained by the dose gradient being located farther from the PTV margin, thereby contributing to the increased robustness of the dose distribution against respiratory phase variations.

These results suggest that, even when tumor position correction using Synchrony functions appropriately, the shape of the dose distribution - particularly the spatial relationship between the PTV and the dose gradient - may influence dose robustness against respiratory phase variations in respiratory tracking radiotherapy. Such dose variations are difficult to identify using conventional dose calculations based solely on a single respiratory-phase CT image, which is typically employed in clinical practice. By performing this type of evaluation at the treatment planning stage, it may be possible to identify potential concerns related to the impact of respiratory phase variations on dose distribution in advance. Furthermore, the results of this evaluation may provide an opportunity to consider plan modifications aimed at improving the robustness of the treatment plan.

This study has several limitations. First, the number of patients was limited to two, and this work should be regarded as an exploratory study presenting representative cases that demonstrated potential dose variations in respiratory tracking radiotherapy. Therefore, caution is required when generalizing the results. Second, different imaging techniques were used for the planning CT and the 4DCT, and thus the influence of differences in image quality on dose calculation could not be fully evaluated. Third, dose variations due to respiration were assessed using several representative respiratory phases extracted from the 4DCT; however, these simulations do not completely reproduce the continuous respiratory motion observed during actual treatment.

In addition, in Synchrony-based treatments, radiation is delivered continuously throughout the entire respiratory cycle, and the clinically relevant tumor dose is evaluated as the cumulative dose integrated over all respiratory phases. Therefore, the phase-specific dose variations demonstrated in this study should be interpreted with caution regarding their direct clinical significance.

Furthermore, although the influence of dose distribution geometry was discussed qualitatively in this study, quantitative evaluations using metrics such as dose fall-off parameters or the gradient index were not performed [19]. Future studies incorporating these quantitative metrics may enable a more objective assessment of dose robustness against respiratory phase variations and further validation of the clinical relevance of the proposed evaluation approach.

## Conclusions

Evaluating the impact of respiratory phase differences on dose distribution in Synchrony-based respiratory tracking radiotherapy, using 4DCT acquired at the time of treatment planning, may be useful as a supplementary, feasibility-level evaluation approach for treatment planning and quality assurance.

The method presented in this study may help identify, in advance, at the planning stage, cases in which respiratory phase variations could potentially compromise target dose coverage.
